# Prevalence of Peri‐Implantitis Around Zirconia Implants: A Systematic Review and Meta‐Analysis of Current Evidence

**DOI:** 10.1111/cid.70176

**Published:** 2026-07-28

**Authors:** Vittorio Moraschini, João Baptista de Moraes, Alice Maria de Oliveira Silva, Alexandre Marques Paes da Silva, Rafael Seabra Louro, Eduardo José Veras Lourenço, Victoria L. Souza Costa, Jamil Awad Shibli

**Affiliations:** ^1^ Department of Oral Surgery, School of Dentistry Fluminense Federal University Niterói Rio de Janeiro Brazil; ^2^ Department of Dental Research, School of Dentistry Veiga de Almeida University Rio de Janeiro Brazil; ^3^ Department of Prosthodontics, School of Dentistry Rio de Janeiro State University Rio de Janeiro Brazil; ^4^ Dental Research Division Universus Veritas Guarulhos University Guarulhos São Paulo Brazil; ^5^ Department of Periodontology, Albert Einstein Israeli Faculty of Health Sciences Albert Einstein Israeli Hospital São Paulo São Paulo Brazil; ^6^ Department of Oral Medicine, Infection, and Immunity Harvard School of Dental Medicine Boston Massachusetts USA; ^7^ Department of Oral Health Sciences, Periodontology KU Leuven and Dentistry, University Hospitals Leuven Leuven Belgium

**Keywords:** dental implants, peri‐implantitis, prevalence

## Abstract

**Background:**

Zirconia implants (ZIs) have emerged as a metal‐free alternative to titanium implants (TIs) because of their favorable esthetic and biological properties. However, the prevalence of peri‐implantitis associated with ZIs and the potential influence of implant material on peri‐implant disease remain unclear.

**Objective:**

To estimate the implant‐level prevalence of peri‐implantitis associated with ZIs and to assess whether implant material influences peri‐implantitis prevalence by comparing ZIs and TIs.

**Methods:**

Electronic searches were conducted in four databases and gray literature sources up to February 2026. Randomized clinical trials (RCTs) and cohort studies evaluating peri‐implantitis associated with ZIs were included. The risk of bias was assessed using the RoB 2 and ROBINS‐I tools. Random‐effects meta‐analyses were performed to estimate the pooled peri‐implantitis prevalence at the implant level. Subgroup analyses according to follow‐up duration and sensitivity analyses were additionally conducted.

**Results:**

Nineteen studies comprising 687 patients and 995 implants were included. The mean follow‐up reported across studies was approximately 49 months. In studies with follow‐up ≤ 3 years, the pooled prevalence of peri‐implantitis associated with ZIs was 2% (95% CI: 0%–6%; *I* ^2^ = 79.3%). Studies with follow‐up > 3 to 5 years demonstrated a pooled prevalence of 2% (95% CI: 0%–8%; *I* ^2^ = 38.9%), whereas studies with follow‐up > 5 years showed a pooled prevalence of 8% (95% CI: 0%–23%; *I* ^2^ = 82.1%). Comparative analysis of five RCTs demonstrated no statistically significant difference in peri‐implantitis prevalence between ZIs and TIs (RR = 1.66; 95% CI: 0.48–5.71; *p* = 0.42). Sensitivity analyses did not identify studies with a critical risk of bias as the primary source of heterogeneity.

**Conclusions:**

Current evidence suggests a low short‐ to mid‐term implant‐level prevalence of peri‐implantitis around ZIs, with higher estimates observed in studies with longer follow‐up. However, the certainty of evidence remains limited because of methodological heterogeneity, variability in diagnostic definitions, inconsistent patient‐level reporting, and the lack of long‐term comparative studies. Limited comparative evidence did not demonstrate a statistically significant difference in peri‐implantitis prevalence between ZIs and TIs.

## 1 Introduction

Dental implant therapy has become one of the most predictable and widely accepted approaches for the rehabilitation of partially and fully edentulous patients. Long‐term evidence from systematic reviews (SRs) with at least 10 years of follow‐up demonstrates that TIs have cumulative survival rates above 94% [1]. These outcomes underscore the biological and mechanical stability of titanium, reinforcing its status as the gold standard material in implant dentistry for over four decades. However, increasing esthetic demands, especially in the anterior region, and concerns related to titanium biocorrosion, release of metal particles, and potential inflammatory responses [2, 3] have stimulated the search for metal‐free alternatives [4].

Zirconia implants (ZIs), typically manufactured from yttria‐stabilized tetragonal zirconia polycrystals (Y‐TZP), have emerged as a promising ceramic option due to their favorable esthetic, mechanical, and biological properties [4]. Zirconia exhibits excellent biocompatibility, high fracture resistance, low plaque affinity, and eliminates soft‐tissue discoloration associated with titanium components [5]. Previous studies report a mean 5‐year survival rate of 97.2% for ZIs, with marginal bone loss (MBL) values around 1.1 mm and stable probing depth (PD), suggesting performance comparable to titanium in mid‐term evaluations [6]. Similar findings have been observed in immediately loaded ZIs, which demonstrate survival rates near 95%, low mechanical complication rates, and favorable soft‐tissue responses [5]. Additional analyses also show that implant design and surface characteristics influence outcomes; for instance, one‐piece ZIs with acid‐etched surfaces tend to exhibit superior clinical performance compared with other configurations [7].

Direct comparisons between TIs and ZIs indicate that both materials achieve similar survival and success outcomes [8, 9, 10]. Recent SRs report lower plaque index (PI) values around ZIs than TIs, while clinical parameters such as bleeding on probing (BOP) and PD show no significant differences between the two materials [11]. Furthermore, evidence has shown that ZIs exhibit MBL comparable to that observed around TIs in the short and medium term [12, 13].

As the use of ZIs expands, understanding the behavior of peri‐implantitis—one of the most impactful biological complications of implant therapy—becomes essential. Peri‐implantitis is defined as a biofilm‐induced inflammatory process affecting peri‐implant tissues, characterized by bleeding and/or suppuration on probing, increased PD, and progressive MBL after prosthetic loading [14]. Recent meta‐analyses estimate an overall peri‐implantitis prevalence of approximately 20% at the patient level and 12%–13% at the implant level, though estimates vary widely depending on diagnostic criteria and follow‐up time [15]. Similar results are reported in broad consensus reviews, where peri‐implantitis prevalence reaches 21%–22% and peri‐implant mucositis affects more than 40% of implant patients, highlighting the major public health relevance of these complications [16].

The etiology of peri‐implantitis is multifactorial, and numerous systemic, behavioral, and local risk factors have been consistently associated with its onset and progression. A recent review reported a higher incidence of peri‐implantitis in the anterior regions of the maxilla and mandible, with a greater prevalence in the maxilla than in the mandible [17]. SRs identify a history of periodontitis, smoking, diabetes mellitus, poor oral hygiene, lack of supportive maintenance, and obesity as key determinants influencing host susceptibility and peri‐implant microbiome imbalance [16]. Additional factors such as excess cement, implant malposition, inadequate keratinized mucosa, occlusal overload, and the presence of titanium particles have also been proposed as risk indicators, although evidence remains limited or inconsistent [18, 19].

Given the increasing clinical adoption of ZIs and the limited number of studies specifically evaluating peri‐implantitis prevalence in these implants, synthesizing the available evidence is crucial to determine whether implant material influences susceptibility to peri‐implant disease. While early observations suggest that zirconia may reduce biofilm accumulation and inflammatory response [4], there is currently no consensus regarding its impact on peri‐implantitis prevalence when compared with titanium.

Although recent SRs have evaluated the clinical performance of ZIs, including survival, MBL, peri‐implant soft‐tissue parameters, and comparisons with TIs, peri‐implantitis prevalence has not been specifically addressed as the primary outcome in a dedicated quantitative synthesis. This distinction is clinically relevant because peri‐implantitis is a cumulative biological complication that cannot be reliably inferred from implant survival, MBL, or isolated peri‐implant clinical parameters. Moreover, estimates of peri‐implantitis prevalence are influenced by substantial variability in diagnostic definitions across studies, including differences in PD thresholds, bleeding and/or suppuration on probing, MBL cutoffs, and timing of assessment. Therefore, a dedicated synthesis focusing on peri‐implantitis prevalence around ZIs is necessary to better interpret the biological complication profile of these implants and to compare the available evidence with TIs.

Therefore, the objective of this SR was to estimate the implant‐level prevalence of peri‐implantitis around ZIs according to follow‐up duration and reported diagnostic criteria and to compare these findings with available data for TIs.

## 2 Methods

### 2.1 Protocol and Registration

The SR protocol was developed in accordance with PRISMA‐P guidelines [20] and registered in the PROSPERO database on December 26, 2025, before the commencement of study selection, including title/abstract screening and full‐text assessment (registration number: CRD420251273821). The final manuscript adhered to the PRISMA reporting recommendations [21].

### 2.2 Focused Questions

1. What is the prevalence of peri‐implantitis in ZIs according to follow‐up duration? (FQ‐1).
2. Is there a difference in the prevalence of peri‐implantitis between ZIs and TIs? (FQ‐2).
3. How do peri‐implant clinical and radiographic parameters (PD, BOP, PI, and MBL) associated with peri‐implantitis vary in ZIs, considering diagnostic criteria and functional loading time? (FQ‐3).

### 2.3 Case Definition

*Peri‐implant mucositis*: was defined as a biofilm‐induced inflammatory condition affecting the peri‐implant mucosa, characterized by the presence of BOP, with or without suppuration, in the absence of progressive MBL beyond initial physiological remodeling after implant placement [22, 23].

*Peri‐implantitis*: was defined as a biofilm‐associated pathological condition occurring in tissues around dental implants, characterized by inflammation of the peri‐implant mucosa, bleeding and/or suppuration on probing, increased PD, and/or mucosal recession, in combination with progressive radiographic MBL compared with previous examinations after prosthetic loading [23].

### 2.4 Eligibility Criteria

As a strategy for the study search and selection process, the eligibility criteria were organized using the PICOS tool:

(P) Population: Patients who received ZIs in function.

(I) Intervention: Prosthetic rehabilitation supported by ZIs (single‐unit, partial, or full‐arch).

(C) Comparison: TIs, when comparative data are available.

(O) Outcomes: Primary outcome: Prevalence of peri‐implantitis associated with ZIs, primarily assessed at the implant level and reported at the patient level when available (FQ‐1). Secondary outcomes: Differences in peri‐implantitis prevalence between ZIs and TIs (FQ‐2), and peri‐implant clinical and radiographic parameters (PD, BOP, PI, and MBL), considering diagnostic criteria and functional loading time (FQ‐3).

(S) Study design: RCTs, controlled clinical trials, and cohort studies.

Studies were eligible only when ZIs were restored and in function for at least 12 months and when peri‐implantitis prevalence, or the explicit presence or absence of peri‐implantitis, was reported. No uniform PD or MBL cutoff was used as an eligibility criterion because peri‐implantitis definitions varied across studies, and no retrospective case definition was imposed. No minimum number of patients or implants was predefined. Therefore, studies were not excluded based on sample size. Animal studies, in vitro studies, case series, case reports, reviews, and studies restricted to specific high‐risk populations were excluded. No restrictions were applied regarding language or publication date.

### 2.5 Literature Search and Study Selection

Two independent reviewers (V.M. and J.B.M.) independently performed the literature search in duplicate. Electronic searches were conducted in four databases (PubMed/MEDLINE, Cochrane Central Register of Controlled Trials, Embase, and Web of Science) to identify studies published up to February 2026. Gray literature was screened using OpenGrey (www.opengrey.eu) and ProQuest Dissertations & Theses (www.theses.com), including both published and unpublished sources. Studies identified by at least one reviewer were advanced to the selection phase. Inter‐reviewer agreement was assessed using Cohen's kappa (*κ*). Disagreements were resolved by consensus or, when necessary, by consultation with a third reviewer (E.J.V.L.) Detailed search strategies and database‐specific terms are presented in Table S1.

### 2.6 Data Items and Extraction

Two reviewers (A.M.O.S. and A.M.P.S.) independently and in duplicate performed manual data extraction from all included studies. When available, the extracted information included authorship, study design, follow‐up duration, number of implants and participants, definitions of peri‐implant risk, prevalence of peri‐implant mucositis and peri‐implantitis at implant and patient levels, implant survival rate, PD, BOP, MBL, and PI, systemic risk indicators, ZIs design, type of prosthesis, implant location, implant system, as well as implant diameter and length. When relevant data were missing, unclear, or inconsistently reported, the corresponding authors of the original studies were contacted by e‐mail to request additional information. If no response was obtained, the available published data were used, and missing information was recorded as not reported.

### 2.7 Risk of Bias Within Studies and Sensitivity Analysis

The risk of bias of the included studies was independently assessed by two reviewers (V.M. and J.M.B.) using the Cochrane Risk of Bias tool version 2 (RoB 2) for RCTs [24] and the ROBINS‐I tool for observational studies [25]. RoB 2 evaluates domains related to the randomization process, deviations from intended interventions, outcome measurement, missing outcome data, selective reporting, and other potential sources of bias. Studies were classified as low risk when all domains were rated as low, high risk when at least one domain was rated as high or multiple domains raised concerns, and as having some concerns when only one domain raised concerns.

For observational studies, ROBINS‐I was used to assess bias due to confounding, participant selection, intervention classification, deviations from intended interventions, missing data, outcome measurement, and selective reporting. Across both tools, domains were categorized as low, moderate, or high risk of bias based on the information reported. No study was excluded based on risk of bias.

Sensitivity analyses were conducted to assess the robustness of the pooled estimates and the influence of individual studies on heterogeneity. A sensitivity analysis according to diagnostic criteria was considered but was not performed because of the limited number of studies within each diagnostic category and the inconsistent reporting of PD, bleeding/suppuration, and MBL thresholds. A leave‐one‐out approach was performed through sequential exclusion of individual studies, followed by reassessment of pooled prevalence estimates and *I* ^2^ values [26]. In addition to the leave‐one‐out analysis, a complementary sensitivity analysis was performed by excluding all studies classified as having a critical risk of bias, and the resulting pooled estimates and heterogeneity values were compared with those from the primary analyses.

### 2.8 Data Analysis and Statistical Methodology

The prevalence of peri‐implantitis was extracted from the included studies as a dichotomous outcome at the implant level and analyzed as proportions. Patient‐level peri‐implantitis prevalence was also extracted and reported descriptively whenever available. Peri‐implantitis prevalence was extracted according to the diagnostic criteria reported by each individual study. Because definitions varied and individual‐level data were not consistently available, no single PD or MBL cutoff was retrospectively imposed across studies. To explore potential sources of heterogeneity and improve the clinical interpretability of the findings, subgroup analysis was performed according to follow‐up duration. A separate comparative analysis of RCTs was also performed. Meta‐analyses were performed using MetaAnalysisOnline.com, a validated web‐based meta‐analysis platform [27]. Pooled proportions and risk ratios (RRs), along with their corresponding 95% confidence intervals (CIs), were calculated to estimate effect sizes. Prediction intervals were additionally computed to enhance clinical interpretability by reflecting the expected range of true effects around the pooled estimates. Given the anticipated clinical and methodological heterogeneity related to differences in study populations, follow‐up periods, and clinical settings, a random‐effects model was applied using the generic inverse variance method. For studies with clustered designs, adjustments were performed in accordance with Cochrane recommendations, applying an intraclass correlation coefficient of 0.07 [5, 28] to account for clustering. When clustering was not addressed in the original reports, sample size corrections were implemented accordingly. Statistical heterogeneity was assessed using the *χ* ^2^ test and interpreted as low (≤ 25%), moderate (> 25% to ≤ 50%), or high (> 50%) [26]. Statistical significance was set at *p* < 0.05. Descriptive analyses, including range, mean, and standard deviation (SD), were conducted for clinical parameter rates and MBL using IBM SPSS Statistics (IBM SPSS Statistics; IBM Corp.).

### 2.9 Risk of Bias Across Studies and Certainty of Evidence

Publication bias was assessed using Egger's regression test in meta‐analyses including more than 10 studies. When fewer than 10 studies were available, publication bias was explored graphically through funnel plots [29]. Sensitivity analyses were conducted to assess the robustness of the pooled estimates and the influence of individual studies on heterogeneity. The certainty of evidence for the main outcomes was assessed using the GRADE approach, considering risk of bias, inconsistency, indirectness, imprecision, and publication bias [30]. The certainty of evidence was classified as high, moderate, low, or very low.

## 3 Results

### 3.1 Study Selection

**FIGURE 1:**
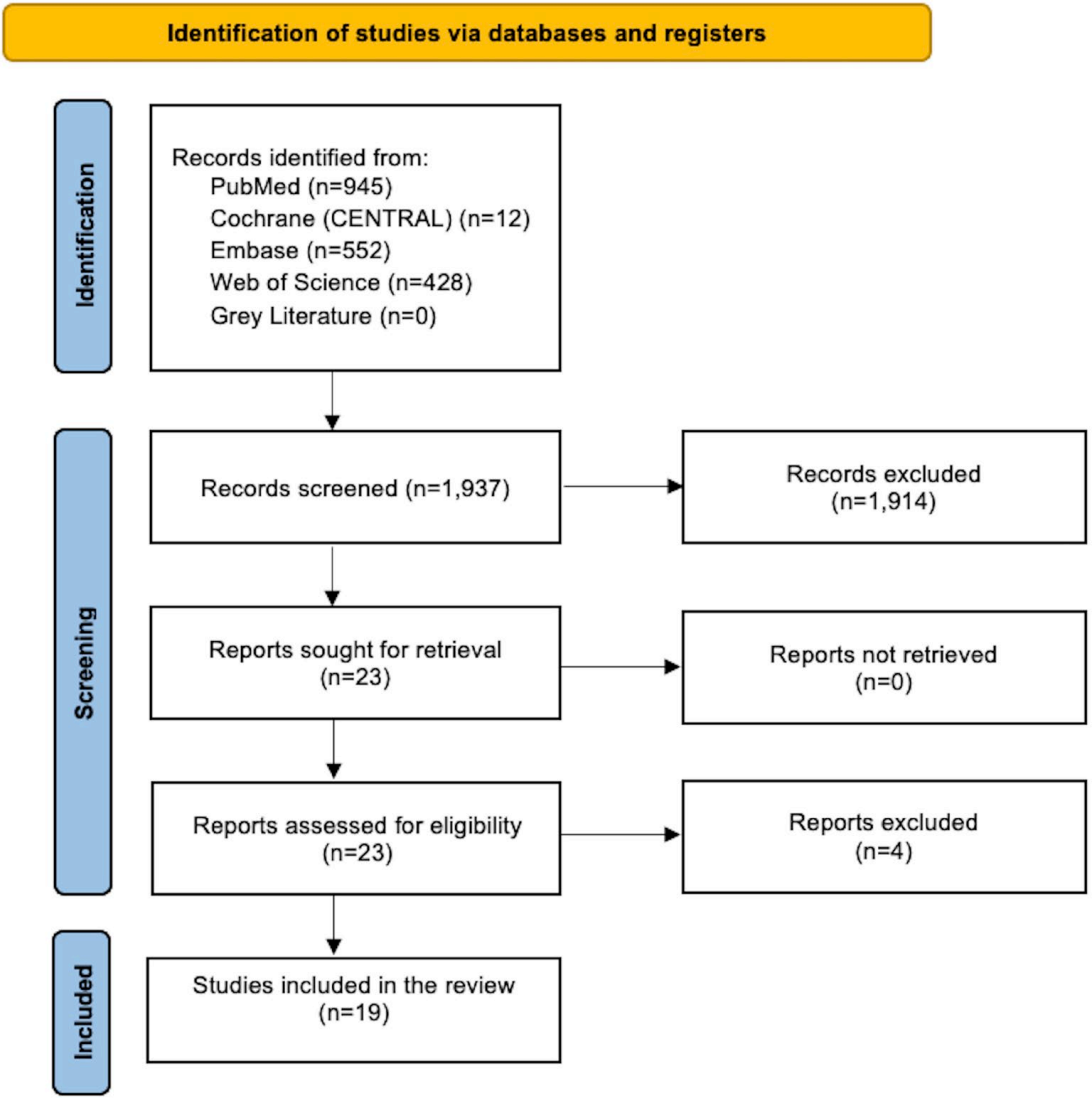
PRISMA flowchart of the screening and selection process.

The database search identified 945 records from MEDLINE/PubMed, 12 from the Cochrane Library, 428 from Web of Science, and 552 from Embase. During the screening of titles and abstracts, 1914 articles were excluded because of duplication or noncompliance with the eligibility criteria (*κ* = 0.97). After full‐text evaluation, an additional four studies were excluded (*κ* = 0.85). A detailed list of excluded studies and the reasons for exclusion is provided in Table S2. Ultimately, 19 studies published between 2015 and 2025 were included in this SR. The study selection process is illustrated in Figure 1.

### 3.2 Basic Characteristics of Included Studies

The main characteristics of the included studies are summarized in Table 1. Overall, the studies comprised 687 patients and 995 dental implants, including 891 ZIs and 104 TIs. The mean follow‐up reported across studies was 49 months, ranging from 12 to 144 months. Regarding study design, five studies were RCTs [8, 9, 10, 34, 37], twelve were prospective cohort studies [31, 32, 35, 36, 38, 39, 40, 41, 43, 44, 45, 46], and two had a retrospective design [33, 42]. Regarding implant design, most studies evaluated one‐piece ZIs, accounting for 73.7% of the included studies (14 out of 19). Two‐piece implant systems were assessed in 21.1% of the studies (*n* = 4), while 5.3% (*n* = 1) investigated both one‐piece and two‐piece designs. Implant survival rates were also extracted and are reported descriptively in Table 1 to contextualize peri‐implantitis prevalence estimates, particularly because implants lost before the final follow‐up assessment may no longer be available for peri‐implantitis diagnosis. Concerning the type of prosthesis, the majority of studies primarily evaluated single crowns, either exclusively or in combination with fixed partial dentures. Several investigations included both prosthetic modalities, whereas overdenture‐supported rehabilitations were rarely reported among the included studies. Finally, ZIs were placed in both the maxilla and mandible across the included studies, with a slight predominance of maxillary placements reported in several cohorts.

**TABLE 1:** Main characteristics of the included studies.

Author (year)	Study design follow‐up time	Number of patients	No. of implants	Peri‐implantitis definition	Prevalence of mucositis at (implant level)	Prevalence of peri‐implantitis (implant level)	Prevalence of peri‐implantitis (patient level)	Implant survival rate	Mean PD (mm)	Mean BOP
Grassi et al. (2015) [31]	Prospective 60 months	17	ZI: 30	− No clinical mobility; − No pain, infection, or suppuration; − No peri‐implant radiolucency; − MBL ≤ 2 mm during the first year	NR	ZI: 1 (3.12%)	NR	ZI: 96.8%	ZI: 2.2	ZI: 0.47%
Holländer et al. (2016) [32]	Prospective 12 months	38	ZI: 94	− No clinical mobility; − No pain, infection, or suppuration; − No clinical mobility; − Progressive MBL	ZI: 14.7%	ZI: 0 (0%)	ZI: 0/38 (0%)	ZI: 100%	ZI: 2.5	ZI: 14.7%
Rodriguez et al. (2018) [33]	Retrospective 25 months	12	ZI: 24	NR*	NR	ZI: 1 (4.1%)	NR	ZI: 92%	NR	NR
Payer et al. (2015) [34]	RCT 24 months	22	ZI: 15 TI: 14	− Implant in position; − Periotest value < +8; − No peri‐implant translucency; − No pain, infection or paraesthesia; − No implant fracture	NR	ZI: 1 (6.25%) TI: 0 (0%)	NR	ZI: 93.3% TI: 100%	NR	ZI: 9.1% TI: 7.4%
Lorenz et al. (2019) [35]	Prospective 68 months	28	ZI: 72	NR*	ZI: 22.1%	ZI: 1 (1.2%)	ZI: 1/28 (3.6%)	ZI: 100%	ZI: 2.57	ZI: 22.1%
Kohal et al. (2018) [36]	Prospective 36 months	65	ZI: 65	− MBL ≤ 2 mm: complete success; − MBL ≤ 3 mm: functional survival	NR	ZI: 2 (3.1%)	ZI: 2/65 (3.1%)	ZI: 90.8%	ZI: 3.3	ZI: 40%
Hassouna et al. (2022) [37]	RCT 60 months	28	ZI: 14 TI: 14	NR*	NR	ZI: 0 (0%) TI: 0 (0%)	ZI: 0/14 (0%) TI: 0/14 (0%)	ZI: 100% TI: 100%	ZI: 3.3 TI: 3.5	NR
Kohal et al. (2023) [38]	Prospective 36 months	27	ZI: 50	− MBL ≤ 2 mm: complete success; − MBL ≤ 3 mm: functional survival	NR	ZI: 12 (23%)	NR	ZI: 98.1%	ZI: 3.35	ZI: 43.7%
Karapataki et al. (2023) [39]	Prospective 144 months	39	ZI: 84	− No clinical mobility; − No pain, infection, or suppuration; − No peri‐implant radiolucency	ZI: 29%	ZI: 8 (9%)	ZI: 0/39 (0%)	ZI: 100%	NR	NR
Kniha et al. (2018) [40]	Prospective 12 months	68	ZI: 90	− No radiographic peri‐implant bone translucency; − Peri‐implant vertical bone loss < 0.2 mm per year; − No implant mobility; − No clinical pain, infection, or nerve damage	NR	ZI: 0 (0%)	ZI: 0/68 (0%)	ZI: 100%	ZI: 1.7	ZI: 0.2
Jung et al. (2016) [41]	Prospective 12 months	60	ZI: 70	NR*	NR	ZI: 0 (0%)	ZI: 0/60 (0%)	ZI: 98.3%	ZI: 3.5	ZI: 89.6%
Borgonovo et al. (2015) [42]	Retrospective 48 months	13	ZI: 19	− No radiographic peri‐implant bone translucency; − MBL ≤ 1.5 mm during the first year and ≤ 0.2 mm annual; − No implant mobility; − No clinical pain, infection, or nerve damage	NR	ZI: 0 (0%)	ZI: 0/13 (0%)	ZI: 100%	ZI: 3	ZI: 0.23%
Balmer et al. (2020) [43]	Prospective 60 months	60	ZI: 70	− No clinical mobility; − No pain; − No peri‐implant radiolucency; − MBL ≤ 2 mm	NR	ZI: 1 (1.79%)	ZI: 1/60 (1.7%)	ZI: 98.4%	ZI: 3.3	ZI: 48%
Brunello et al. (2022) [44]	Prospective 111 months	30	30	− Presence of bleeding and/or suppuration; − PD ≥ 6 mm; − MBL ≥ 3 mm	ZI: 44.8%	ZI: 0 (0%)	ZI: 0/30 (0%)	ZI: 96.7%	ZI: 3.0	ZI: 12.9%
Zuercher et al. (2024) [10]	RCT 12 months	40	ZI: 38 TI: 38	− Absence of erythema, BOP, swelling and suppuration	ZI: 76.2%	ZI: 2 (5.3%) TI: 1 (2.6%)	ZI: 2/40 (5.0%) TI: 1/40 (2.5%)	ZI: 100% TI: 100%	ZI: 2.8 TI: 2.8	ZI: 34.2% TI: 25.8%
da Silva et al. (2024) [45]	Prospective 12 months	50	ZI: 63	NR*	NR	ZI: 0 (0%)	ZI: 0/50 (0%)	ZI: 98.5%	NR	NR
Henao et al. (2024) [9]	RCT 60 months	30	ZI: 16 TI: 14	− Presence of BOP (peri‐implant mucositis); Increased PPD and MBL (peri‐implantitis)	ZI: 12.5% TI: 21.4%	ZI: 3 (18.7%) TI: 2 (14.2%)	ZI: 3/16 (18.7%) TI: 2/14 (14.2%)	ZI: 100% TI: 100%	ZI: 3.09 TI: 3.51	ZI: 13.1% TI: 19.3%
de Beus et al. (2024) [8]	RCT 12 months	48	ZI: 24 TI: 24	NR*	NR	ZI:0 (0%) TI: 0 (0%)	ZI: 0/24 (0%) TI: 0/24 (0%)	ZI: 96% TI: 100%	ZI: 3.2 TI: 3.2	NR
Kohal et al. (2025) [46]	Prospective 120 months	12	ZI: 23	− Positive BOP; − Suppuration; − PD ≥ 6 mm; − MBL ≥ 2 mm	ZI: 33.3%	ZI: 8 (33.3%)	NR	ZI: 59.2%	ZI: 3.94	ZI: 68.7

Author (year)	Mean MBL (mm)	Mean PI	Systemic risk indicators	ZI implant design	Type of prosthesis implant location	Implant system implant diameter and length (mm)	Approximate adjustment for clustering	Design effect adjustment (for implants)
Grassi et al. (2015) [31]	ZI: 1.23	ZI: 40%	Light smokers (< 10 cigarettes/day)	1‐piece	SC (Cemented) Max (81%) Mand (19%)	WhiteSKY (Bredent Medical) 8, 10, 12, 14, 16 × 3.5, 4, 4.5	No	1.06
Holländer et al. (2016) [32]	NR	ZI: 19.7%	Systemically healthy patients	1‐piece	SC (Cemented) Max (50%) Mand (50%)	Z‐Look 3 implant system (Z‐Systems) NR	No	1.13
Rodriguez et al. (2018) [33]	ZI: 0.3	ZI: 12.5%	Smoker (1) History of cancer (2)	1‐piece (10) 2‐piece (14)	SC (Cemented) Max (83.3%) Mand (16.7%)	Z‐Look 3 implant system (Z‐Systems) NR	Yes	None
Payer et al. (2015) [34]	ZI: 1.48 TI: 1.43	ZI: 19.3% TI: 16%	Systemically healthy patients	2‐piece	SC (Cemented) Max (29.1%) Mand (70.9%)	Ziterion vario Z (ZI) Ziterion vario T (TI) 10, 11.5, 13 × 4.0	No	1.03
Lorenz et al. (2019) [35]	ZI:1.2	ZI: 25%	Systemically healthy patients	1‐piece	SC (Cemented) Max (45.7%) Mand (54.3%)	Z‐Look 3 implant system (Z‐Systems) 8 to 14 × 3.25 to 5	No	1.14
Kohal et al. (2018) [36]	ZI: 1.45	ZI: 18%	Smokers (4) Bruxism (4)	1‐piece	SC (Cemented) Max (20%) Mand (80%)	ZiUnite (Nobel Biocare) 10, 13, 16 × 4.3, 5.0	No	1.00
Hassouna et al. (2022) [37]	ZI: 1.77 TI: 1.8	NR	Light smokers (< 10 cigarettes/day)	1‐piece	SC (Cemented) Max (100%)	Customized implant 12 × 3.7, 4.5	No	1.00
Kohal et al. (2023) [38]	ZI: 2.16	ZI: 18.2%	Systemically healthy patients	1‐piece	FDP Max (81.5%) Mand (18.5)	ZiUnite (Nobel Biocare) 10, 13, 16 × 4.3, 5.0	No	1.07
Karapataki et al. (2023) [39]	NR	NR	Smokers ≤ 30 cigarettes per day	2‐piece	SC and OVD (Cemented and screw‐retained) Max (32.4%) Mand (67.6%)	Customized implant 9, 10, 11, 13 × 4.1, 4.5, 4.7, 5, 5.3	No	1.09
Kniha et al. (2018) [40]	ZI: 3.09	ZI: 0%	Systemically healthy patients	1‐piece	SC (Cemented) Max/Mand	PURE Ceramic (Straumann) 8, 10, 12 × 4, 5.5	No	1.02
Jung et al. (2016) [41]	ZI: 0.78	ZI: 21.4%	Systemically healthy patients	1‐piece	SC and FDP (Cemented) Max (32.3%) Mand (67.7%)	Customized implant 8, 10, 12, 14 × 4.0, 4.5, 5.5	No	1.01
Borgonovo et al. (2015) [42]	ZI: 2.1	ZI: 12.7%	Smokers of up 10 cigarettes per day	1‐piece	SC and FDP (Cemented) Max (75%) Mand (25%)	WhiteSKY (Bredent Medical) NR	No	1.04
Balmer et al. (2020) [43]	ZI: 0.7	ZI: 26.2%	Smokers ≤ 10 cigarettes per day	1‐piece	SC and FDP (Cemented) Max (32.4%) Mand (67.6%)	Ceramic.implant (Vita) 8, 10, 12, 14 × 4.0, 4.5, 5.0	No	1.01
Brunello et al. (2022) [44]	NR	ZI: 17.2%	Systemically healthy patients	2‐piece	SC (Cemented) Max (33%) Mand (67%)	Patent (Zircon Medical) 9, 11, 13 × 4.5, 5.0	No	1.00
Zuercher et al. (2024) [10]	ZI: 0 TI: 0.3	NR	Smokers ≤ 15 cigarettes per day	1‐piece	SC (Cemented) Max (62.5%) Mand (37.5%)	PURE Ceramic (Straumann) 8, 10, 12 × 4.1	No	1.07
da Silva et al. (2024) [45]	ZI: 0.25	NR	Systemically healthy patients	2‐piece	SC (Cemented and screw‐retained) Max (55.4%) Mand (44.6%)	ZI Ceramic Implant (Neodent) 10, 11, 13 × 3.75, 4.3	No	1.02
Henao et al. (2024) [9]	ZI: 2.35 TI: 2.76	ZI: 50% TI: 86%	Smokers ≤ 10 cigarettes per day	1‐piece	SC (Cemented) Max (100%)	PURE Ceramic (Straumann) and Standard Plus Narrow Neck CrossFi (Straumann) 8, 10, 12 × 3.5	No	1.00
de Beus et al. (2024) [8]	ZI: 0.01 TI: 0.09	NR	Systemically healthy patients	1‐piece	SC (Cemented) Max (100%)	PURE Ceramic (Straumann) and Ti Bone Level (Straumann) NR	No	1.00
Kohal et al. (2025) [46]	ZI: 1.61	ZI: 54.6%	Systemically healthy patients	1‐piece	FPD (Cemented) Max (58.3%) Mand (41.7%)	ZiUnite (Nobel Biocare) 10, 13, 16 × 4.3, 5.0	No	1.07

*Note:* BOP, bleeding on probing; FPD, fixed partial dentures; Mand, mandible; Max, maxilla; MBL, marginal bone loss; NR, not reported; OVD, overdentures; PD, probing depth; PI, plaque index; RCT, randomized controlled trial; SC, single crown; TI, titanium; ZI, zirconia.

* NR in the peri‐implantitis definition column indicates that no formal case definition was provided by the original study; however, the presence or absence of peri‐implantitis was explicitly reported.

### 3.3 Synthesis of Results

#### 3.3.1 Prevalence of Peri‐Implantitis (FQ‐1)

##### 3.3.1.1 At the Patient Level

Patient‐level peri‐implantitis prevalence, when available, is reported descriptively in Table 1. Because patient‐level data were inconsistently reported and could not be reliably derived from implant‐level events in studies including multiple implants per patient, a separate patient‐level meta‐analysis was not performed.

##### 3.3.1.2 At the Implant Level

**FIGURE 2:**
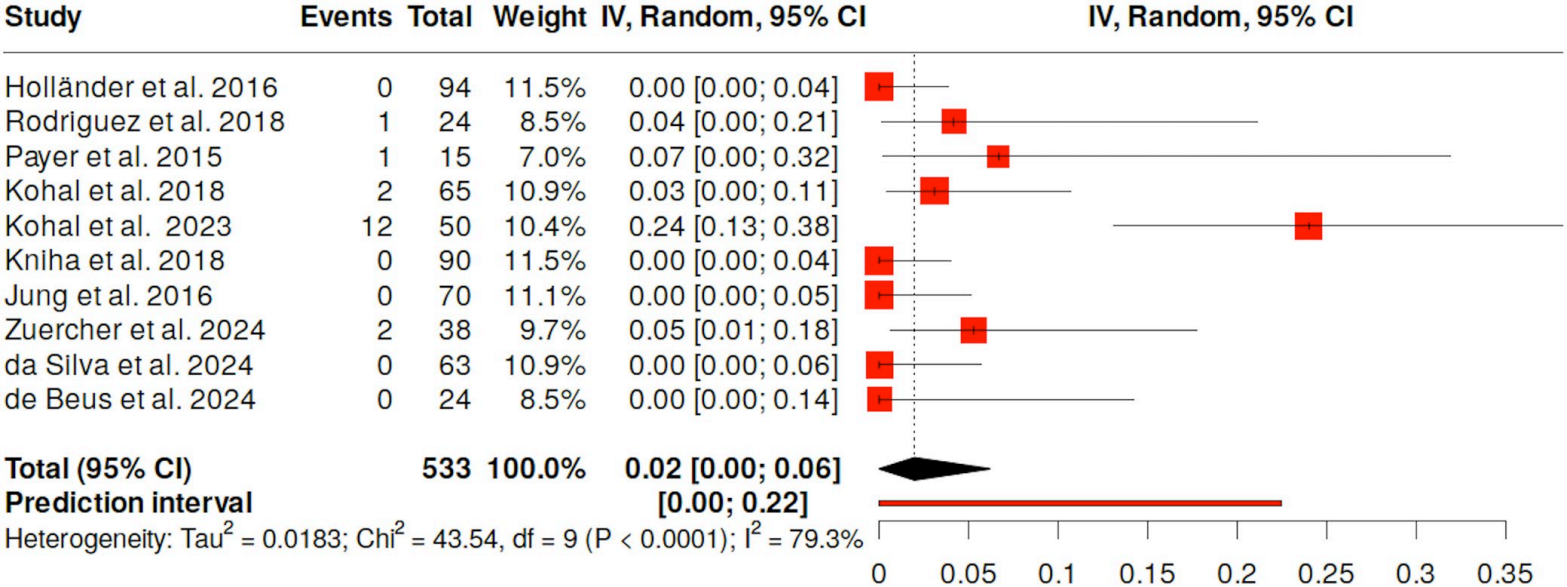
Forest plot of the pooled prevalence of peri‐implantitis in zirconia implants with follow‐up ≤ 3 years.

Across the included studies evaluating ZI, with a mean follow‐up time of 49 months, the reported prevalence of peri‐implantitis at the implant level showed substantial variability, ranging from 0% to 33.3%. When pooled using a random‐effects meta‐analysis, the overall prevalence of peri‐implantitis in ZIs in a follow‐up period of ≤ 3 years [8, 10, 32, 33, 34, 36, 38, 40, 41, 45] was estimated at 2% (95% CI: 0%–6%), with a wide prediction interval ranging from 0% to 22% (Figure 2). A high degree of heterogeneity was observed among studies (*I* ^2^ = 79.3%; *p* < 0.0001), indicating substantial inter‐study variability (Figure 2).

**FIGURE 3:**
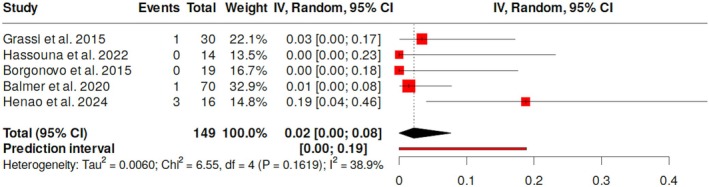
Forest plot of the pooled prevalence of peri‐implantitis in zirconia implants with follow‐up > 3 to 5 years.

**FIGURE 4:**
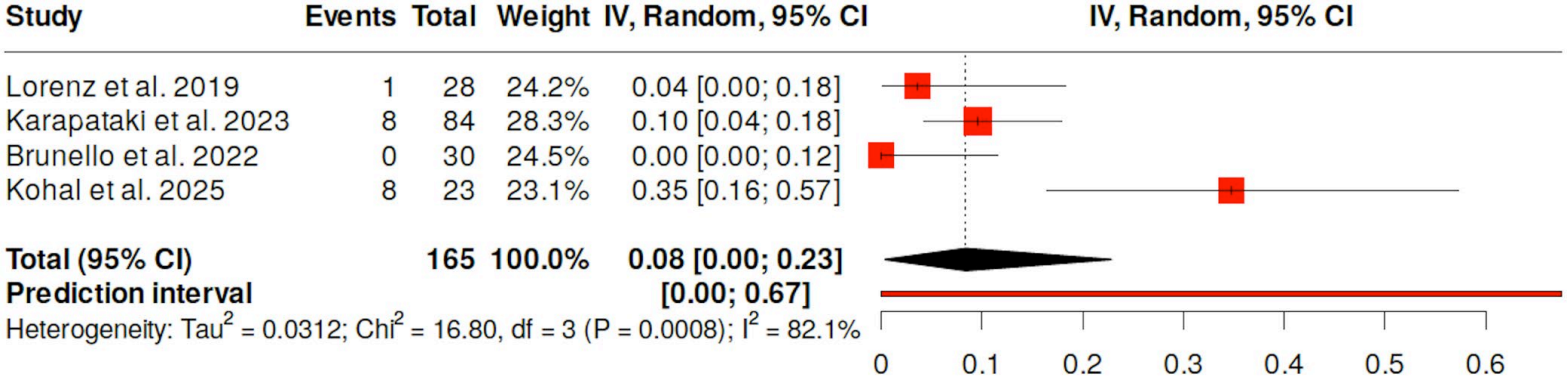
Forest plot of the pooled prevalence of peri‐implantitis in zirconia implants with follow‐up > 5 years.

For studies with a follow‐up period of > 3 to 5 years [9, 31, 37, 42, 43], the pooled prevalence of peri‐implantitis in ZIs was estimated at 2% (95% CI: 0%–8%), with a prediction interval ranging from 0% to 19% (Figure 3). A low‐to‐moderate degree of heterogeneity was observed among studies (*I* ^2^ = 38.9%; *p* = 0.16), suggesting relatively consistent prevalence estimates within this follow‐up category. For studies with a follow‐up period of > 5 years [35, 39, 44, 46], the pooled prevalence of peri‐implantitis in ZIs increased to 8% (95% CI: 0%–23%), with a very wide prediction interval (0%–67%) (Figure 4). A high degree of heterogeneity was observed among studies (*I* ^2^ = 82.1%; *p* = 0.0008), indicating substantial inter‐study variability within long‐term follow‐up investigations.

#### 3.3.2 Prevalence of Peri‐Implantitis Between Zirconia and Titanium Implants (FQ‐2)

**FIGURE 5:**
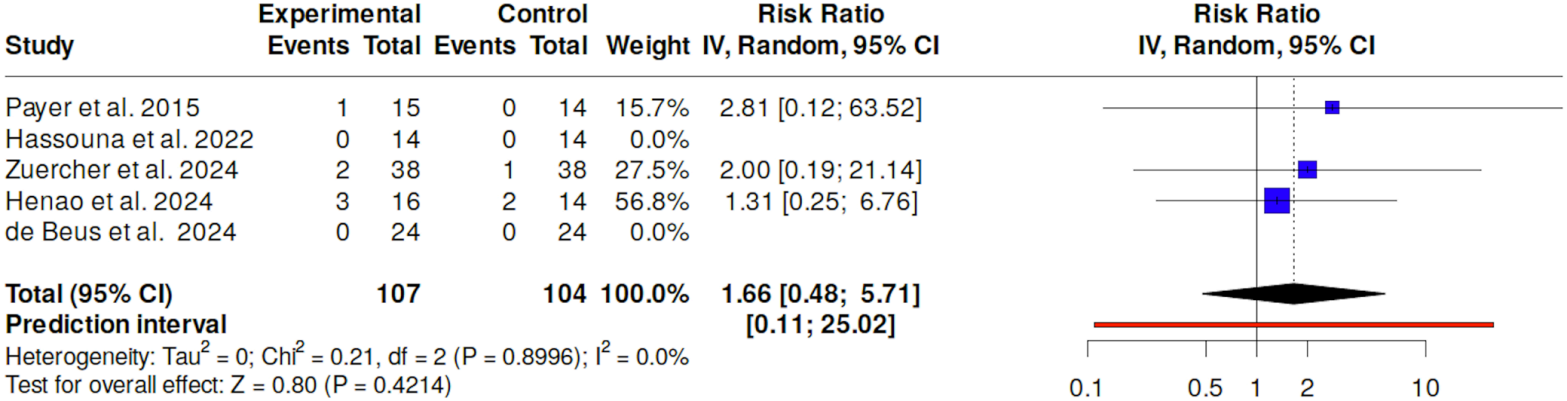
Forest plot comparing the risk of peri‐implantitis between zirconia and titanium implants.

Five RCTs [8, 9, 10, 34, 37] comparing ZIs and TIs were included in the meta‐analysis, comprising 109 ZIs and 106 TIs. The pooled random‐effects analysis showed no statistically significant difference in peri‐implantitis prevalence between materials (RR = 1.66; 95% CI: 0.48–5.71; *p* = 0.42). No between‐study heterogeneity was detected (*I* ^2^ = 0%; *p* = 0.89), and the prediction interval ranged from 0.11 to 25 (Figure 5).

#### 3.3.3 Clinical Parameters (FQ‐3)

Meta‐analysis of clinical parameters was not feasible because of inconsistent reporting methods, variable outcome definitions, and substantial methodological heterogeneity across the included studies. Among studies evaluating ZIs, mean PD values ranged from 1.7 to 3.94 mm, with an overall mean of 2.98 mm. BOP demonstrated marked inter‐study variability, with reported values ranging from 0.2% to 89.6%. MBL values ranged from 0 to 3.09 mm, with an average of 1.28 mm. Similarly, PI values showed considerable variability among studies, ranging from 0% to 54.6% (Table 2).

**TABLE 2:** Descriptive statistics of clinical and radiographic parameters assessed around zirconia implants.

Parameter	No. of studies	Mean	Median	Range
PD (mm)	15	2.98	3.09	1.7–3.94
BOP (%)	14	28.4	18.4	0.2–89.6
MBL (mm)	16	1.28	1.34	0–3.09
PI (%)	14	23.9	19.5	0–54.6

Abbreviations: BOP, bleeding on probing; MBL, marginal bone loss; PD, probing depth; PI, plaque index.

### 3.4 Risk of Bias Within Studies and Sensitivity Analysis

Risk of bias was assessed using RoB 2 for RCTs and ROBINS‐I for observational studies. Overall, RCTs were judged to present “some concerns,” mainly due to deviations from intended interventions and outcome measurement, reflecting the difficulty of blinding in implant dentistry. Allocation concealment was insufficiently reported in some trials, while missing outcome data were generally limited. No randomized study was judged to be at high risk of bias.

Most observational studies showed a “moderate risk of bias,” primarily related to confounding due to the absence of randomized comparators and limited adjustment for prognostic factors. Four studies [32, 33, 40, 42] were judged to be at “critical risk of bias” because of the absence of control groups and substantial methodological limitations. Detailed risk of bias assessments are presented in Tables S3 and S4.

In addition to the leave‐one‐out sensitivity analysis, a complementary analysis was performed by simultaneously excluding all studies classified as having a critical risk of bias [32, 33, 40, 42]. This analysis produced pooled prevalence estimates comparable to those of the primary analyses and did not meaningfully reduce heterogeneity, indicating that these studies did not exert a substantial collective influence on the pooled estimates. Overall, heterogeneity appeared to be more strongly associated with clinical and methodological differences among the included studies, particularly differences in follow‐up duration, diagnostic criteria, and reported peri‐implantitis prevalence.

### 3.5 Risk of Bias Across Studies and Certainty of Evidence

**FIGURE 6:**
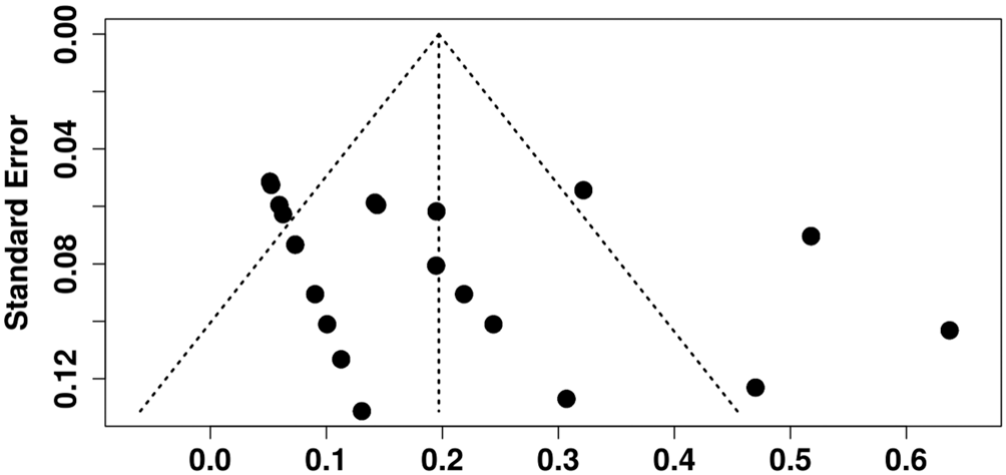
Funnel plot for assessment of publication bias.

Publication bias was assessed using funnel plot inspection and Egger's regression test. Visual examination of the funnel plot did not demonstrate substantial asymmetry. In line with this observation, Egger's test did not provide statistical evidence of small‐study effects or publication bias (intercept: 2.49; 95% CI: −0.58 to 5.55; *t* = 1.588; *p* = 0.13), with the CI crossing zero. These findings should be interpreted with caution, as the power of asymmetry tests is limited in meta‐analyses of prevalence and when a moderate number of studies are included. Overall, no indication of substantial publication bias was identified (Figure 6). The certainty of evidence ranged from low to very low across outcomes. The evidence for implant‐level peri‐implantitis prevalence was mainly downgraded because of risk of bias, heterogeneity in diagnostic criteria, and imprecision. The comparative evidence between ZIs and TIs was rated as low, mainly because of imprecision related to the small number of events and wide CIs. The Summary of Findings is presented in Table S5.

## 4 Discussion

### 4.1 Synthesis of the Evidence

This SR aimed to estimate the prevalence of peri‐implantitis in ZIs and to determine whether implant material influences the risk of developing peri‐implantitis when compared with TIs. The null hypothesis—that there is no difference in peri‐implantitis prevalence between ZIs and TIs—was not rejected, as the pooled analysis of RCTs demonstrated no statistically significant difference between materials. These findings suggest that implant material alone does not appear to be a decisive determinant of peri‐implantitis development, reinforcing the multifactorial nature of peri‐implant diseases [14, 16]. Regarding the first focused question, the pooled prevalence estimates of peri‐implantitis in ZIs remained low in short‐ and mid‐term follow‐up periods, although associated with a wide prediction interval. This result suggests that ZIs may achieve favorable peri‐implant health outcomes, comparable to those reported for TIs in long‐term epidemiological studies [15, 16], while highlighting that peri‐implantitis risk is strongly influenced by follow‐up duration and patient‐related factors rather than implant material per se [17, 18]. However, the pooled prevalence estimates should be interpreted with caution and should not be viewed as definitive epidemiological estimates. The available evidence is limited by heterogeneous peri‐implantitis definitions, variable follow‐up periods, inconsistent reporting of patient‐level data, and differences in implant design, prosthetic configuration, and maintenance protocols. Therefore, the present findings are better interpreted as a synthesis of the currently available evidence rather than as a precise estimate of the true prevalence of peri‐implantitis around ZIs.

The prevalence of peri‐implantitis associated with ZIs varied substantially across studies, ranging from 0% to 33.3%. This wide dispersion reflects heterogeneity in follow‐up time, peri‐implantitis case definitions, implant systems, and study designs. The subgroup analyses according to follow‐up duration demonstrated a progressive increase in peri‐implantitis prevalence over time. While short‐term studies showed very low prevalence estimates, studies with follow‐up periods exceeding 5 years reported higher prevalence values and greater heterogeneity. These findings support the concept that peri‐implantitis is a cumulative and time‐dependent condition influenced by prolonged functional loading and patient‐related factors [47]. Additionally, the higher heterogeneity observed in long‐term studies likely reflects differences in diagnostic criteria, maintenance protocols, and clinical characteristics among cohorts.

Differences in maintenance protocols, supportive peri‐implant care, implant systems, and diagnostic criteria may have influenced the reported prevalence estimates. Regular supportive therapy and plaque control may reduce the progression from peri‐implant mucositis to peri‐implantitis, whereas inconsistent maintenance may increase variability across studies [47, 48]. In addition, implant design, prosthetic configuration, surface characteristics, and implant–abutment connection may affect hygiene accessibility, biofilm accumulation, and peri‐implant tissue response [49]. Finally, differences in peri‐implantitis definitions, including PD thresholds, bleeding and/or suppuration on probing, MBL cutoffs, and reference radiographs, may have directly influenced case classification and prevalence estimates [15, 23].

Peri‐implant mucositis, recognized as an early and reversible inflammatory condition and a potential precursor of peri‐implantitis [49], was poorly and inconsistently reported among the included studies. Most investigations did not include mucositis as a predefined outcome, and when reported, prevalence estimates showed substantial variability and were not systematically correlated with subsequent peri‐implant bone loss. This lack of standardized reporting limits the interpretation of disease progression around ZIs and hampers the identification of early biological changes preceding peri‐implantitis. Likewise, the potential influence of prosthetic design on peri‐implantitis prevalence could not be adequately assessed. Although prosthesis‐related factors—such as splinting, emergence profile, and hygiene accessibility—may affect peri‐implant tissue stability [48, 50], none of the included studies were specifically designed to evaluate the association between prosthetic type and peri‐implant disease. The predominance of single crowns and the limited reporting of overdentures or stratified analyses according to prosthetic configuration precluded meaningful comparisons between prosthetic modalities.

The comparative findings between ZIs and TIs should be interpreted cautiously. Although the pooled analysis of RCTs did not demonstrate a statistically significant difference in peri‐implantitis prevalence between materials, the available evidence was based on a limited number of trials, most with short‐ to mid‐term follow‐up and few peri‐implantitis events. Therefore, the absence of a statistically significant difference should not be interpreted as definitive evidence of equivalence between implant materials. Longer‐term randomized studies using standardized peri‐implantitis definitions are needed to determine whether material‐related differences may influence peri‐implant disease over time.

Clinical parameters associated with peri‐implant health, including PD, BOP, PI, and MBL, showed marked inter‐study variability. Mean PD values generally remained within clinically acceptable ranges [51], while BOP and PI varied widely, likely reflecting differences in maintenance protocols and oral hygiene standards across cohorts. MBL values were generally modest and comparable to those reported for TIs in longitudinal studies [1, 23], suggesting stable peri‐implant bone conditions in most ZIs cohorts.

Microbiological data were available from only two observational studies. Holländer et al. [32] reported that ZIs exhibited lower plaque accumulation than natural teeth, while overall bacterial load was comparable, with a higher relative detection of selected periodontitis‐associated species. Similarly, Lorenz et al. [35], in a long‐term follow‐up study, observed comparable total bacterial colonization between ZIs and natural teeth. However, red‐complex pathogens were more frequently detected around implants. Importantly, these microbiological differences were not consistently associated with adverse clinical or radiographic outcomes, suggesting that the presence of specific pathogens alone may be insufficient to trigger peri‐implant disease in the absence of host susceptibility or unfavorable local conditions [14, 16].

### 4.2 Strengths and Limitations

This SR presents several methodological strengths. A comprehensive search strategy without language or publication date restrictions was applied, minimizing selection and publication bias. The inclusion of both randomized and observational studies allowed for a broad synthesis of available evidence, while cluster‐adjusted analyses and prediction intervals enhanced the robustness and clinical interpretability of prevalence estimates [26]. However, several limitations must be acknowledged. Substantial heterogeneity was observed across studies, largely driven by inconsistent peri‐implantitis definitions, variable follow‐up durations, and heterogeneous reporting of clinical parameters. Although a sensitivity analysis according to diagnostic criteria was considered, it was not feasible because of the limited number of studies within each diagnostic category and the inconsistent reporting of clinical and radiographic thresholds. Despite the adoption of contemporary case definitions in recent studies [23], older or surrogate success criteria were frequently used, limiting comparability. Additionally, most included studies evaluated one‐piece ZIs, restricting the generalizability of findings to two‐piece systems. The influence of overdenture‐supported rehabilitations could not be separately evaluated because only a very limited number of included studies involved overdentures, and peri‐implantitis outcomes were not consistently stratified according to prosthetic design.

Another limitation of the present SR is that the meta‐analysis was primarily conducted at the implant level, since patient‐level prevalence data were inconsistently reported across the included studies. This limitation reflects a broader issue in the current ZIs literature, in which most investigations preferentially report implant‐based outcomes rather than patient‐based prevalence estimates. Consequently, direct comparisons with epidemiological studies evaluating peri‐implant diseases at the patient level should be interpreted with caution.

### 4.3 Clinical Implications and Future Recommendations

From a clinical perspective, the findings of this review suggest that ZIs may be considered a viable alternative to TIs in appropriately selected patients, particularly in esthetically demanding situations or in patients with concerns regarding metal exposure. However, this interpretation should be cautious, as peri‐implantitis risk appears to be primarily influenced by patient‐related factors, maintenance protocols, follow‐up duration, prosthetic design, and diagnostic criteria rather than implant material alone.

Future research should prioritize well‐designed, long‐term comparative studies directly comparing ZIs and TIs using standardized peri‐implantitis definitions and uniform outcome reporting. Future trials and cohort studies should report peri‐implantitis at both implant and patient levels, clearly describe maintenance and supportive peri‐implant care protocols, and provide detailed information on implant design, prosthetic configuration, and diagnostic thresholds. Such standardization is necessary to improve comparability across studies and to determine whether implant material, implant design, or prosthetic factors influence peri‐implantitis prevalence over time.

## 5 Conclusions

Within the limitations of the available evidence, current data suggest a low short‐ to mid‐term implant‐level prevalence of peri‐implantitis around ZIs. However, prevalence estimates tended to increase in studies with longer follow‐up, supporting the concept that peri‐implantitis is a cumulative and time‐dependent condition. The certainty of evidence remains limited because of methodological heterogeneity, variability in diagnostic definitions, inconsistent patient‐level reporting, and the limited availability of long‐term comparative studies. Limited comparative data from RCTs did not demonstrate a statistically significant difference in peri‐implantitis prevalence between ZIs and TIs. Therefore, these findings should be interpreted cautiously until more standardized, long‐term, patient‐level comparative evidence becomes available.

## Author Contributions

Conceptualization and Study Design: V.M., E.J.V.L., J.A.S. Data Analysis and Interpretation: V.M., E.J.V.L., R.S.L. Drafting of the Manuscript: V.M., E.J.V.L., J.A.S. Data Collection: J.B.M., A.M.O.S., A.M.P.S., V.L.S.C. Critical Review and Revision: V.M., E.J.V.L., R.S.L., J.A.S. Funding Acquisition: V.M., J.A.S.

## Funding

Supported by the Fundação Carlos Chagas Filho de Amparo à Pesquisa do Estado do Rio de Janeiro—FAPERJ, funded V.M. (grant # 204395/2025), and the National Council for Scientific and Technological Development, Brazil (CNPq), funded J.A.S. (CNPq # 314479/2023‐6 and CNPq # 408905/2025‐5).

## Ethics Statement

The authors have nothing to report.

## Conflicts of Interest

The authors declare no conflicts of interest.

## Supporting information


**Table S1:** Search strategy.
**Table S2:** Excluded studies.
**Table S3:** Cochrane risk‐of‐bias tool for RCTs (RoB 2).
**Table S4:** ROBINS‐I risk‐of‐bias tool for non‐randomized studies.
**Table S5:** Summary of findings and certainty of evidence according to the GRADE approach.

## Data Availability

The data that support the findings of this study are available from the corresponding author upon reasonable request.
