# Supplementary material for: Prevalence of Peri‐Implantitis Around Zirconia Implants: A Systematic Review and Meta‐Analysis of Current Evidence
Source: Clin Implant Dent Relat Res. 2026 Jul 28;28(4):e70176. doi: 10.1111/cid.70176 (PMC13408551; doi:10.1111/cid.70176)
Supplement: Supplementary file 1 — Table S1: Search strategy. Table S2: Excluded studies. Table S3: Cochrane risk‐of‐bias tool for RCTs (RoB 2). Table S4: ROBINS‐I risk‐of‐bias tool for non‐randomized studies. Table S5: Summary of findings and certainty of evidence according to the GRADE approach. [file CID-28-0-s001.docx]

**SUPPLEMENTAL MATERIALS**

**Supplemental Table 1.** Search strategy

| **Database** | **Search strategy** |
| --- | --- |
| PubMed / MEDLINE | ("Zirconia Implants"[Mesh] OR "zirconia implant*" OR "zirconium dioxide implant*" OR "ceramic implant*" OR "Y-TZP implant*") AND ("Peri-Implantitis"[Mesh] OR peri-implantitis OR periimplantitis OR "peri-implant disease*" OR "peri-implant inflammation") AND (prevalence OR epidemiology OR incidence OR "clinical parameter*" OR "marginal bone loss" OR probing depth OR bleeding) |
| Embase | ('zirconia implant'/exp OR 'zirconium dioxide implant' OR 'ceramic implant' OR 'yttria stabilized zirconia' OR Y-TZP) AND ('periimplantitis'/exp OR periimplantitis OR 'peri implant disease' OR 'peri implant inflammation') AND (prevalence OR epidemiology OR incidence OR 'clinical parameter' OR 'marginal bone loss' OR 'probing depth' OR 'bleeding on probing') |
| Web of Science | TS=(("zirconia implant*" OR "zirconium dioxide implant*" OR "ceramic implant*" OR "Y-TZP") AND (peri-implantitis OR periimplantitis OR "peri-implant disease*" OR "peri-implant inflammation") AND (prevalence OR epidemiology OR incidence OR "clinical parameter*" OR "marginal bone loss")) |
| Cochrane CENTRAL | (zirconia implant* OR ceramic implant* OR zirconium dioxide implant*) AND (peri-implantitis OR periimplantitis OR peri-implant disease*) AND (prevalence OR epidemiology OR marginal bone loss) |
| OpenGrey | zirconia implant* AND peri-implantitis |
| ProQuest Dissertations & Theses | ("zirconia implant*" OR "ceramic implant*") AND (peri-implantitis OR periimplantitis) |

**Supplemental Table 2.** Excluded studies.

| **N** | **Reference** | **Reason** |
| --- | --- | --- |
| 1 | Schwarz F, John G, Hegewald A, Becker J. Non-surgical treatment of peri-implant mucositis and peri-implantitis at zirconia implants: a prospective case series. J Clin Periodontol 2015;42:783−788. | Focus only on treatment |
| 2 | Balmer M, Spies BC, Vach K, Kohal RJ, Hämmerle CHF, Jung RE. Three-year analysis of zirconia implants used for single-tooth replacement and three-unit fixed dental prostheses: A prospective multicenter study. Clin Oral Implants Res 2018;29:290−299. | Duplicate study |
| 3 | Bienz SP, Hilbe M, Hüsler J, Thoma DS, Hämmerle CHF, Jung RE. Clinical and histological comparison of the soft tissue morphology between zirconia and titanium dental implants under healthy and experimental mucositis conditions-A randomized controlled clinical trial. J Clin Periodontol 2021;48:721−733. | Induced mucositis |
| 4 | Siddiqi A, Kieser JA, De Silva RK, Thomson WM, Duncan WJ. Soft and Hard Tissue Response to Zirconia versus Titanium One-Piece Implants Placed in Alveolar and Palatal Sites: A Randomized Control Trial. Clin Implant Dent Relat Res. 2015;17:483-96. | Peri-implantitis was not specifically assessed or explicitly reported |

**Supplemental Table 3.** Cochrane risk-of-bias tool for RCTs (RoB 2).

| **Study** | **Randomization process** | **Deviations from intended interventions** | **Missing outcome data** | **Measurement of the outcome** | **Selection of the reported result** | **Overall risk of bias** |
| --- | --- | --- | --- | --- | --- | --- |
| Payer et al., 2014/2015 | Some concerns | Some concerns | Low risk | Some concerns | Some concerns | Some concerns |
| Hassouna et al., 2022 | Low risk | Some concerns | Low risk | Some concerns | Some concerns | Some concerns |
| Zuercher et al., 2023 | Low risk | Some concerns | Low risk | Some concerns | Low risk | Some concerns |
| Ruiz Henao et al., 2024 | Low risk | Some concerns | Some concerns | Some concerns | Some concerns | Some concerns |
| de Beus et al., 2024 | Low risk | Some concerns | Low risk | Some concerns | Some concerns | Some concerns |

**Supplemental Table 4.** ROBINS-I risk-of-bias tool for non-randomized studies.

| **Study** | **Bias due to confounding** | **Selection of participants** | **Classification of interventions** | **Deviations from intended interventions** | **Missing data** | **Measurement of outcomes** | **Selection of reported result** | **Overall risk of bias** |
| --- | --- | --- | --- | --- | --- | --- | --- | --- |
| Balmer et al., 2020 | Moderate | Low | Low | Low | Moderate | Moderate | Moderate | Moderate |
| Borgonovo et al., 2015 | Critical | Moderate | Low | Low | Moderate | Moderate | Moderate | Critical |
| Brunello et al., 2022 | Moderate | Moderate | Low | Low | Moderate | Moderate | Moderate | Moderate |
| Da Silva et al., 2024 | Moderate | Moderate | Low | Low | Low | Moderate | Moderate | Moderate |
| Grassi et al., 2015 | Moderate | Moderate | Low | Low | Moderate | Moderate | Moderate | Moderate |
| Holländer et al., 2016 | Critical | Moderate | Low | Low | Low | Moderate | Moderate | Critical |
| Jung et al., 2015 | Moderate | Moderate | Low | Low | Moderate | Moderate | Moderate | Moderate |
| Karapataki et al., 2023 | Moderate | Moderate | Low | Low | Low | Moderate | Moderate | Moderate |
| Kniha et al., 2018 | Critical | Moderate | Low | Low | Low | Moderate | Moderate | Critical |
| Kohal et al., 2023 | Moderate | Moderate | Low | Low | Moderate | Moderate | Moderate | Moderate |
| Kohal et al., 2017 | Moderate | Moderate | Low | Low | Moderate | Moderate | Moderate | Moderate |
| Lorenz et al., 2019 | Moderate | Moderate | Low | Low | Moderate | Moderate | Moderate | Moderate |
| Rodriguez et al., 2018 | Critical | Moderate | Low | Low | Moderate | Moderate | Moderate | Critical |
| Kohal et al., 2025 | Moderate | Moderate | Low | Low | Moderate | Moderate | Moderate | Moderate |

**Supplemental Table 5 -** Summary of findings and certainty of evidence according to the GRADE approach.

| **Outcome** | **Studies / implants** | **Effect estimate** | **Certainty of evidence** | **Main reasons for downgrading** |
| --- | --- | --- | --- | --- |
| Peri-implantitis prevalence in ZIs, ≤3 years | 11 studies / 579 implants | 1% (95% CI: 0–5%) | Very low | Risk of bias, high inconsistency, imprecision, variability in diagnostic criteria |
| Peri-implantitis prevalence in ZIs, >3 to 5 years | 6 studies / 221 implants | 2% (95% CI: 0–5%) | Low | Risk of bias, imprecision, variability in diagnostic criteria |
| Peri-implantitis prevalence in ZIs, >5 years | 3 studies / 137 implants | 10% (95% CI: 0–32%) | Very low | High inconsistency, serious imprecision, few studies |
| ZIs versus TIs for peri-implantitis prevalence | 6 RCTs / 155 ZIs and 158 TIs | RR = 1.13 (95% CI: 0.33–3.91) | Low | Some concerns in risk of bias and serious imprecision |

GRADE, Grading of Recommendations Assessment, Development and Evaluation; ZIs, zirconia implants; TIs, titanium implants; CI, confidence interval; RR, risk ratio; MBL, marginal bone loss; PD, probing depth; BOP, bleeding on probing. Certainty of evidence was rated as high, moderate, low, or very low according to the GRADE domains: risk of bias, inconsistency, indirectness, imprecision, and publication bias. Reasons for downgrading are described in the final column.
